# How Suicides Are Made

**Published:** 1890-07-26

**Authors:** 


### The Doctor's Pulpit.

### HOW SUICIDES ARE MADE.

"When all the blandishments of life are gone,
The coward sneaks to death, the brave live on."

It seems certain that more men and women commit suicide now than formerly. Children very seldom destroy themselves, though they have less fear of death than grown-up persons, and a much feebler apprehension of the possible terrors of Hades. The fact that children do not commit suicide suggests one reason why men and women do. Children usually have the cup of health well filled, and where they are at all in happy circumstances the cup very frequently overflows. A smile and a contented look on a child's face indicate full average well-being; a romp, a shout, and a ringing laugh are all signs that the cup has flowed over. The child must necessarily have a surplus of health, because, in addition to maintaining his usual strength, his bones and muscles have to grow, and his brain and other internal organs must be developed. The exuberant feeling of well-being in the child makes his positive pleasures delightful, and reduces his griefs to a minimum, both of severity and of duration. The idea of suicide, therefore, never occurs to him, except, perhaps, when he has permitted himself to indulge in an unusually bad fit of rage or disappointment. Then for five minutes, or even for a quarter of an hour, he may long to punish his "unkind" mother or his "stern" father for their denial of some ardent wish by hanging or drowning himself. The fact that he would not be alive to see what he considers their well-deserved agony is usually sufficient to prevent him from putting his desperate thought into practice.

Just as a child does not think of suicide because his health is too buoyant, so does the grown-up person often look longingly towards it because his health is too depressed. It is impossible to separate the spirits from the health, the body from the mind. A certain amount of water will float a ship; a full flowing tide and a steady wind will bear her gallantly along at full speed. But if water fail and wind go down her sails flap idly against the masts, and she sinks a helpless hulk into the imprisoning sand. It is exactly so with the physical health. In favourable circumstances it is like the full flowing tide and the high wind; and life is then a prosperous and delightful voyage from shore to shore. But when health fails completely and permanently the power of physical and intellectual movement is impaired or gone, the spirits sink to their lowest ebb, and to a certain order of character life becomes absolutely miserable, and death is looked upon as the only possible relief.

From this point of view the cause of suicide, like the cause of madness, is entirely physical, and the lines we have quoted at the top of the page are unscientific and unjust. But in this, as in so many other of the affairs of life, a sufficiently careful observation of facts shows that the cause is seldom quite simple; that, indeed, as a rule, many causes have been in operation, and that for a long period, before the final culmination of suicide has been reached. At the actual moment of self-destruction probably no single person in the world's history has ever been quite himself; and, therefore, at that moment responsibility and guilt are alike to be denied. But no man allows the plea of the confirmed drunkard, that he cannot help himself, to be an absolution from guilt. There was a time when he could have helped himself, and when the voice of duty imperatively demanded that he should do so. Having weakly and wickedly yielded to temptation then, his final destruction by drink was rendered inevitable. The case of the suicide is often parallel with that of the drunkard. It is in the earlier stages of failing health, sleeplessness, mental anxiety, and so on, that a man should resolutely deal with himself. We say "should deal with himself" because, although it is the doctor's duty to supply his patient with information, advice, and guidance, he cannot furnish him with a clear intelligence and a firm will. It is here, in fact, that the modern doctor is so grossly ill-used a man. He sees distinctly the state of his patient's health, and the cause of his mental suffering. He tells the unfortunate man, as precisely and plainly as language can convey his judgment, that worries are to be promptly put away, that the thoughts are to be turned into other channels, and that complete rest is to be secured. If the patient will not, or thinks he cannot, obey these directions, that is his misfortune, or it may be his fault. But in no sense can the doctor be made responsible.

It is commonly believed that the tendency to suicide, like the tendency to madness, runs in families, and that is no doubt true. But the strongest-minded and clearest-headed man in the world has the possibility of suicide in him. On the other hand, the disposition to madness and suicide, which is so decided a characteristic of some families is, in many cases, easily to be kept at bay by resolution and intelligence on the part of particular individuals. So that, in most cases, if the story of a suicide be read from the very beginning, the full responsibility must be placed upon the victim himself. In our own time the pressure of highly civilized environment urges men in the direction of brain weariness and so of disgust with life. But it is to be borne in mind that no man is compelled to enter into the keenest competitions of his age. The brain is fairly mature before the age of twenty-five; and before that age few educated men are married, and fewer still are irrevocably committed to a particular calling or way of life. A young man of average intelligence is then quite able to judge his own intellectual force and staying power, and he is also able to take into consideration the history of his family and his own inherited tendencies. It is incumbent upon him at that stage to take stock of his mental and physical resources exactly as he takes stock of his capital. If his available money amount to no more than one or two thousand pounds he would consider himself a madman were he to embark in a business requiring a capital of half a million. But is he not just as much a madman if, with a mind of merely average powers, he enters upon a line of life requiring an intellect of the strongest and clearest order and mental endurance of the most persistent kind? A young man acting thus invites brain worry, invites chronic dyspepsia, invites sleeplessness; throws the door wide open for the entrance of all the physiological foes that destroy health and drive sanity out of the home. Many a man of business knows well what it is to be beaten. There are bankrupts by the score who have fought gallantly until the last drop of their financial blood has been shed, and who have been compelled in the long run to haul down their flag. It is exactly so in many cases with the mental and bodily health. A man fights, and toils, and labours until the last penny of his physiological capital is paid away and gone. Then he is beaten and throws down his arms, and despair stalks in and takes possession of the citadel of his manhood. It is at this point that some men refuse to live any longer. They will not submit to so great and final a defeat, and so they resolve upon the desperate venture of extinguishing life's candle for ever.

When a man has arrived at this pass his kindly fellow-men pity rather than blame him; and they humbly trust that Divine Providence judges as they judge. But pity cannot alter the scientific facts of the case; and those facts are that the suicide entered upon a course of life for which he ought to have known that he was unfit, and that he persisted in it when his judgment assured him that there was no hope of success. There are, then, two things which a stern medical science says to all men. The first is: Do not enter upon a calling or a way of life for which your mental, physical, and financial resources are not reasonably adequate. The second is: If you have entered upon such a way of life, and find yourself being steadily and irresistibly pushed down towards final and hopeless defeat, rein in your steed before you go any further, and at any and every cost, turn back and make a fresh start upon different lines. This is difficult, you say? Yes! Life is sometimes difficult; so difficult as to seem impossible. But all the possible calamities of a man's career, who is in dire straits, must be placed side by side and accurately measured. When this measurement is done, it is seen that two of those possible calamities are absolute mountains in size compared with all the others. Those two are madness and suicide. If it be true that self-destruction is on the increase, and that every human being has in himself the possibility of that catastrophe, the subject demands the earnest consideration of every strenuous and heavily-burdened man.

